# Evaluation of Salusin-α and Salusin-β Levels in Human Saliva Samples from Patients with Gingivitis and Periodontitis: A Cross-Sectional Study

**DOI:** 10.3390/biomedicines14020346

**Published:** 2026-02-02

**Authors:** Fatma Tuba Akdeniz, Zerrin Barut, Ahmet Mert Nalbantoglu, Turgay İsbir

**Affiliations:** 1 Department of Genetics and Bioengineering, Faculty of Engineering and Natural Sciences, Istanbul Okan University, Istanbul 34959, Türkiye; 2 Department of Basic Medical Sciences, Faculty of Dentistry, Antalya Bilim University, Antalya 07190, Türkiye; 3 Department of Periodontology, Faculty of Dentistry, Süleyman Demirel University, Isparta 32200, Türkiye; 4 Department of Molecular Medicine, Institute of Health Science, Yeditepe University, Istanbul 34755, Türkiye

**Keywords:** gingivitis, periodontitis, salusins, saliva, biomarkers, salusin-α, salusin-β

## Abstract

**Background**: Gingivitis and periodontitis are progressive inflammatory diseases affecting the tissues surrounding the teeth; gingivitis involves reversible gingival inflammation, whereas periodontitis is a more advanced condition characterized by irreversible tissue destruction, including clinical attachment and alveolar bone loss. Salusin-α and salusin-β are inflammation-related polypeptides that may reflect periodontal inflammatory burden; however, salivary data in periodontal diseases are lacking. This study aimed to evaluate the salivary salusin-α and salusin-β levels in individuals with gingivitis and periodontitis. **Methods**: Saliva samples were collected from a total of 80 systemically healthy non-smoker individuals classified into three groups: gingivitis (*n* = 27), stage III grade B periodontitis (*n* = 27), and healthy participant (*n* = 26) based on the 2017 Periodontal Classification criteria. Salusin-α and salusin-β levels in saliva were quantified using enzyme-linked immunosorbent assays (ELISA). Statistical analysis utilized one-way ANOVA, Student’s *t*-test, and Receiver Operating Characteristic (ROC) curve analysis. **Results**: Compared to the healthy group, salivary levels of salusin-α and salusin-β were found to be significantly elevated in periodontitis groups (*p* < 0.05), not gingivitis (*p* > 0.05); moreover, the increase in both markers was significantly greater in the periodontitis group than in the gingivitis group (*p* < 0.05). **Conclusions**: Our finding suggests that salusins play a role in the inflammatory processes of periodontal diseases. The increase in salusin-α and salusin-β levels in the periodontitis suggests that these parameters may serve as biomarkers.

## 1. Introduction

Periodontal diseases are inflammatory conditions that include hard and soft tissues supporting the teeth, characterized by the inflammation of periodontal tissues [1]. Gingivitis is an inflammation that occurs in the gingival tissues and usually develops as a result of the gradual and continuous accumulation of bacteria [2]. This inflammatory process is caused by the microbial accumulation of dental plaque on the tooth surface, further exacerbated by the disruption of the balance between the host immune system and bacterial flora [1,2]. In addition, local and systemic factors that increase plaque accumulation contribute to the development and progression of inflammation [3]. Periodontitis is a chronic inflammatory condition triggered similarly by biofilm, which causes the breakdown of the periodontium and leads to the gradual loss of both periodontal and alveolar bone support surrounding the teeth, potentially resulting in tooth loss [2]. Despite similar levels of exposure to microbial plaque, the immune response to periodontal inflammation may vary among individuals. This is consistent with the current classification of periodontal diseases, which acknowledges that genetic predispositions, along with systemic and environmental factors, can substantially influence the inflammatory process [4]. In periodontal disease, salivary levels of disease-related biomarkers—such as lectins, C-reactive protein (CRP), proteases, cystatins, and cytokines—provide valuable information, particularly regarding the severity of inflammatory conditions [5,6]. Due to the complex bacterial composition of gingivitis or periodontitis, the host immune system mounts a strong inflammatory response with the release of various biomarkers [5]. While these mediators are involved in host defense and the inflammatory changes may be reversible with appropriate periodontal therapy, their prolonged elevation is associated with irreversible tissue destruction, particularly clinical attachment loss (CAL) and alveolar bone loss [7,8]. Crucially, the chronic nature of periodontal diseases means that the continuous inflammatory burden within the oral cavity does not remain localized. This condition serves as a source of low-grade systemic inflammation, allowing the translocation of bacterial components and inflammatory mediators into the systemic circulation [1,7].

The role of biomarkers in the early diagnosis and monitoring of periodontal diseases is becoming increasingly important [9]. While traditional clinical and radiographic assessments generally reveal changes at more advanced stages of the disease, biomarkers allow detection at earlier, even subclinical, stages [4]. This not only accelerates the diagnostic process but also enables the development of personalized treatment approaches. On the other hand, monitoring the inflammatory process using materials such as saliva, which can be easily obtained non-invasively from patients, may be relatively easier [5,9].

Salusins, endogenous biopeptides consisting of 20–28 amino acids, salusin-α and salusin-β, are expressed in the brain, immune cells, endothelium, muscle and nervous system, plasma, kidney, and bone marrow [10,11]. Salusins are closely associated with oxidative stress and inflammatory responses [12]. It has been shown that salusin-β increases the levels of proinflammatory cytokines, including interleukin (IL)-6, IL-8, and IL-18, and TNF-α concurrently, and decreases the expression of the IL-1 receptor antagonist, which serves as the natural antagonist of IL-1 [13,14]. Salusin-β, with its proinflammatory properties, promotes foam cell formation, whereas salusin-α exerts anti-atherogenic effects by inhibiting foam cell formation and preventing vascular sclerosis [15]. Given that the underlying mechanisms driving both periodontal destruction and systemic pathologies involve lipid dysregulation, endothelial dysfunction, and inflammation [9,16,17], it is hypothesized that the imbalance between salusins may also be reflected within the periodontal disease process. The systemic role of salusins in inflammation is well-established [10,13,14]; however, there is a distinct gap in the literature concerning their specific involvement in periodontal disease. To date, to the best of the authors’ knowledge, there is no study that has analyzed salusin-α and salusin-β levels in individuals with gingivitis or periodontitis.

Consequently, given the lack of available data in the literature, the study aimed to evaluate salivary levels of salusin-α and salusin-β in gingivitis and periodontitis. The null hypotheses of the study were that [1] levels of salusin-α and salusin-β would not be statistically different in gingivitis compared to healthy patients, and [2] levels of salusin-α and salusin-β would not be statistically different in periodontitis compared to healthy patients.

## 2. Materials and Methods

### 2.1. Study Population

This study was approved by the local ethics committee (Antalya Bilim University Rectorate, Non-Interventional Research Ethics Committee of the Faculty of Health Sciences, decision no: 2025-107, decision date: 4 July 2025). The research was conducted in accordance with the ethical principles outlined in the Declaration of Helsinki. All participants were provided with detailed information about the study, and written informed consent was obtained from each individual.

**Figure 1:**
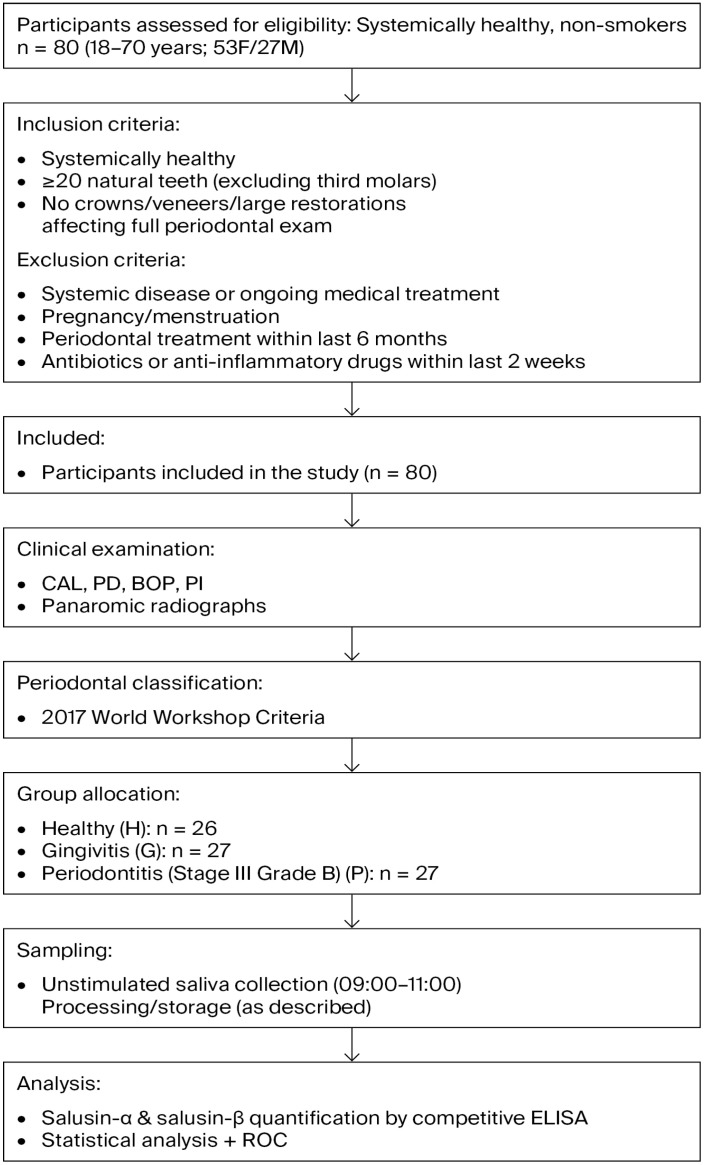
Flow chart illustrating the study design, participant selection, inclusion and exclusion criteria, group allocation, and salusin measurement procedure.

To detect a required minimum sample size, based on a pilot study that included 10 samples for each of three groups (healthy, gingivitis, and periodontitis), an alpha error of 0.05, statistical power of 0.95, and an effect size of 0.60, a minimum sample size of 20 (considering laboratory losses, 6–7 samples were added to each group) samples per group was calculated using the G*Power 3.1.9.2 software. The study workflow is illustrated in Figure 1.

### 2.2. Study Design

In this study, a total of 80 saliva samples were used to assess salusin-α and salusin-β levels in gingivitis and periodontitis. The concentrations of salusin-α and salusin-β in the saliva were measured using a competitive enzyme-linked immunosorbent assay (ELISA) method characterized by high sensitivity and validated specificity. The study participants consisted of 80 systemically healthy, non-smoking individuals (53 females and 27 males, aged between 18 and 70 years) in order to minimize potential confounding factors.

Inclusion criteria required participants with systemic health, not undergoing any medical treatment, to have more than 20 natural teeth (excluding third molars), and to have no dental crowns, veneers, or large restorations that could compromise the accuracy of a full periodontal examination. Exclusion criteria were individuals with any systemic disease or who were currently receiving medical treatment, pregnant, menstruating, had received periodontal treatment within the previous six months, or had taken any local or systemic antibiotics or anti-inflammatory drugs in the last two weeks.

Participants were allocated into three groups according to periodontal health status: group H, healthy individuals; group G, individuals with gingivitis; and group P, individuals with periodontitis. Group H (6 males, 20 females; mean age 26.2 years) consisted of individuals with clinically healthy periodontium. These participants showed no clinical attachment loss (CAL), probing depth (PD) of 3 mm or less, and whole-mouth bleeding on probing (BOP) below 10%. Group G (10 males, 17 females; mean age 29.2 years) included individuals diagnosed with gingivitis. In this group, no CAL or radiographic bone loss was observed, PD was 3 mm or less, and BOP was 10% or higher. Group P (11 males, 16 females; mean age 44.1 years) comprised patients diagnosed with Stage III Grade B periodontitis. At least two non-adjacent sites had PD between 3–4 mm or CAL of 5 mm or more, with radiographic bone loss ranging from 15% to 35% or extending to the middle third of the root. Panoramic radiographs were used to assess alveolar bone loss.

The diagnostic criteria for gingivitis and healthy periodontal conditions were based on the 2017 World Workshop classification of periodontal and peri-implant diseases and conditions. For each participant, clinical periodontal measurements such as CAL, PD, and plaque index (PI) were documented. The PI was determined by applying the Silness and Löe [18] scoring system at six different locations on each tooth using a periodontal probe.

### 2.3. Saliva Sampling

For the study, unstimulated saliva was acquired. Included individuals were trained to brush their teeth at home in the morning before coming to the hospital, and to avoid drinking, eating, or chewing gum 1 h before the sampling of the saliva. Saliva was obtained between 09:00 and 11:00 in the morning at a similar clinical room temperature by a plastic cup and transferred to centrifuge tubes using sterile syringes. The saliva samples were centrifuged at 1000× *g* for 15 min at 4 °C. Following sample processing, the supernatants were immediately aliquoted and rapidly frozen at −80 °C without any delay and were thawed only once prior to ELISA analysis.

### 2.4. The Procedure of ELISA

The ELISA kits were kept at +4 °C following the manufacturer’s storage guidelines. Before initiating the analysis, all reagents were brought to room temperature (18–25 °C), and the preparation of reagents was performed in accordance with the instructions provided by the manufacturer. After the saliva samples had thawed, they were centrifuged at 3000× *g* for 15 min at +4 °C. In this study, saliva concentrations of salusin-α and salusin-β were quantified using commercially available competitive ELISA kits (Human Salusin Alpha ELISA Kit -ABT1522Hu and Human Salusin Beta ELISA Kit- ABT2549Hu). Each microplate provided in the kits was pre-coated with an antibody specific to the respective salusin peptide.

According to the assay principle, salusin peptides in the standards or samples compete with immobilized peptides on the plate for binding to a biotinylated detection antibody. Following incubation, unbound substances and excess antibodies are removed through washing steps. Avidin–Horseradish Peroxidase (HRP) is then added to each well to initiate a colorimetric enzymatic reaction. The final optical density is measured to determine the peptide concentration.

During the experiment, a volume of 50 μL of diluted standards, blank controls, or saliva samples was dispensed in duplicate into each well. This was followed by the immediate addition of 50 μL of the biotin-labeled antibody working solution. The microplate was then covered and incubated at 37 °C for 30 min.

Following the incubation period, each well was rinsed three times with 300 μL of 1× wash buffer, and excess liquid was removed using absorbent paper. Subsequently, 100 μL of Avidin–HRP conjugate was added to all wells, and the plate was incubated once more at 37 °C for 30 min. The washing steps were then repeated using the same protocol.

Afterward, 100 μL of Tetramethylbenzidine (TMB) substrate was dispensed into each well, and the plate was kept in the dark at 37 °C for around 15 min, ensuring the incubation time did not exceed 30 min. Once adequate color development was observed, 50 μL of stop solution was added to each well, causing the color to shift from blue to yellow.

OD values were immediately measured at 450 nm using a preheated microplate reader (SYNERGY/HTX Multi-Mode Reader). Concentrations (pg/mL) of salusin-α and salusin-β were calculated using the corresponding standard curves. In competitive ELISA, inverse OD values (1/absorbance) were calculated to determine analyte concentrations.

Salusin-α detection range is 78.13–5000 pg/mL salusin-β detection range is 31.25–2000 pg/mL. The intra-assay coefficient of variation (CV) for the salusin-α and salusin-β ELISA kits was reported by the manufacturer as <10%.

### 2.5. Statistical Analysis

All statistical analyses were performed using the SAS 9.4 software package. Data normality was evaluated through the Kolmogorov–Smirnov test. To compare the study groups, one-way ANOVA post hoc Tukey’s HSD was conducted. Relationships between continuous variables were examined using Pearson correlation analysis, while associations between categorical variables were evaluated with Chi-square tests.

Moreover, Receiver Operating Characteristic (ROC) curve analysis was applied to assess the sensitivity and specificity of salusin-α and salusin-β. These curves illustrate the relationship between sensitivity (true positive rate) and 1-specificity (false positive rate) across a range of threshold values. The area under the curve (AUC) serves as an indicator of the test’s diagnostic capability—values closer to the upper left corner of the plot reflect greater diagnostic accuracy.

Optimal cutoff values were identified using the Youden Index (calculated as Sensitivity + Specificity − 1). ROC analysis was conducted for both salusin-α and salusin-β to determine their diagnostic utility. A *p* < 0.05 was regarded as statistically significant.

## 3. Results

The descriptive statistics and comparison results of sex and age in the gingivitis, periodontitis, and healthy groups are presented in Table 1. A Chi-square analysis was performed to examine the relationship between sex and the study groups, and no significant correlation was found between sex and the three groups (*p* > 0.05), which shows that the males and females are evenly distributed across the study groups.

In contrast, age differed significantly between the three study groups (*p* = 0.0001). To determine the source of the significant differences among the groups, Tukey’s HSD post hoc test demonstrated that the gingivitis and healthy groups had similar age averages, which were lower than those of the periodontitis group.

**Figure 2:**
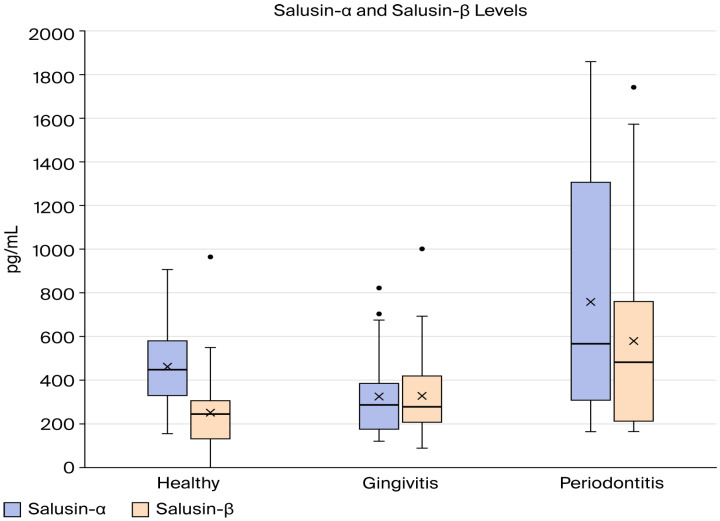
Comparison of salusin-α and salusin-β levels between the study groups.

Table 2 summarizes the descriptive statistics and comparison and correlation results of salusin-α and salusin-β between group H, group G, and group P, and the results are illustrated graphically in Figure 1. As illustrated in Table 2, there was a statistically significant variation in mean salusin-α levels between the study groups (*p* < 0.05). According to the Tukey HSD post hoc test, group H and group G had similar salusin-α levels, while group P had elevated salusin-α levels compared to healthy (*p* = 0.024). In addition, there is a statistical difference between group G and group P (*p* = 0.001). The one-way ANOVA results indicated a statistically significant difference in mean salusin-β levels among the three study groups (*p* < 0.05). According to the Tukey HSD post hoc analysis, group P had the highest average salusin-β level, followed by the gingivitis group, with the healthy group showing the lowest value (Figure 2).

To assess the relationship between salusin-α and salusin-β within each study group, a correlation analysis was conducted, as demonstrated in Table 2. A strong, statistically significant positive correlation was observed between salusin-α and salusin-β in both the gingivitis and periodontitis groups (gingivitis: r = 0.8131, *p* = 0.0001; periodontitis: r = 0.8043, *p* = 0.0001). In contrast, no significant association was found between these two markers in the healthy group (*p* > 0.05).

ROC curve analysis was performed to specify the sensitivity and specificity for different threshold values of salusin-α and salusin-β to assess their potential in predicting disease status. The optimal cut-off points for both markers, along with their corresponding sensitivity and specificity, are reported. Furthermore, an empirical ROC curve reflecting these results was generated using a non-parametric approach in the SAS software.

**Figure 3:**
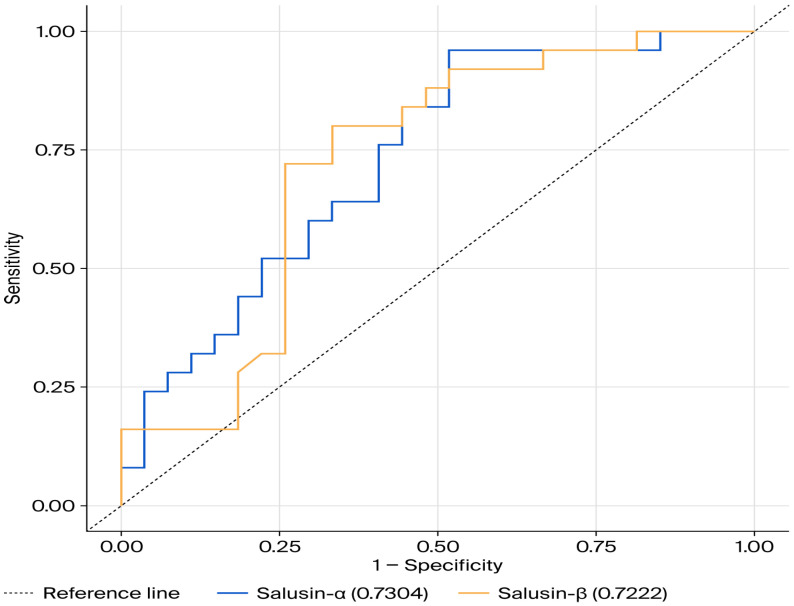
ROC Curve for salusin-α and salusin-β.

Table 3 presents the results of the ROC curve analysis for salusin-α and salusin-β, with the corresponding ROC curves shown in Figure 3. The ROC curve analysis for salusin-α resulted in a 95% confidence interval of 0.594–0.867, an area under the ROC curve (AUC) value of 0.730 (*p* < 0.05). The ROC analysis for salusin-β resulted in a 95% confidence interval of 0.579–0.865, an AUC value of 0.722, and a *p*-value of 0.002 (Table 3, Figure 3). The ROC curve for salusin-α and salusin-β indicates its predictive capacity to distinguish individuals with disease from healthy individuals.

## 4. Discussion

This study, for the first time in the literature, showed that salivary salusin-α and salusin-β levels were increased in periodontitis, not gingivitis. To the best of our knowledge, salusin-α and salusin-β levels have not been previously investigated in gingival crevicular fluid (GCF) and/or systemic circulation in patients with periodontitis. The first null hypothesis was accepted, while the second one was rejected. Periodontal disease is a worldwide notable public health issue, impacting not only oral health but also systemic inflammation and overall health status [19]. Current evidence indicates that periodontal diseases are a modifiable risk factor associated with various systemic diseases. This underscores the importance of biomarker-based research aimed at elucidating the systemic effects of periodontal diseases and highlights the need to prioritize these conditions more in global health policies [7].

Salusin-α and salusin-β are peptides associated with inflammatory processes and are considered potential markers of systemic inflammation [20]. Previous studies have reported that these peptides exhibit different effects, particularly in diseases such as atherosclerosis and hypertension [21]. For example, salusin-α is known to have atheroprotective properties, while salusin-β is known to trigger the production of reactive oxygen species (ROS), and its inhibition reduces ROS production [22]. Additionally, salusin-beta has been reported to play an active role in endothelial cell inflammation [13,23]. Capability of microorganisms originating from the subgingival biofilm to enter the bloodstream and infect endothelial cells supports the molecular link between periodontal disease and systemic atherosclerotic processes [24].

The inhibition of foam cell formation induced by salusin-β by salusin-α in atherosclerosis and the promotion of vascular smooth muscle cell proliferation by salusin-β indicate that these two peptides have opposing effects in the development of cardiovascular diseases [14,25,26]. In our study, the increased levels of both salusin-α and salusin-β in individuals with periodontitis suggest that these two parameters may have tissue-specific regulatory mechanisms. It is considered that these differences may arise from biochemical or genetic factors and should be clarified through further research.

The increased serum salusin-α levels observed in other systemic inflammatory diseases such as rheumatoid arthritis and Behçet’s disease support the potential role of these peptides in rheumatic pathologies [27,28]. The fact that periodontal diseases share similar immune-inflammatory imbalance mechanisms with rheumatoid arthritis and Behçet’s disease [29,30] suggests that salusins may be involved in these common inflammatory pathways. Changes in salusin levels in both disease groups provide important clues about the molecular basis of the relationship between periodontal and systemic inflammation.

The synthesis of salusins in immune-related cells and their active involvement in systemic inflammatory processes suggest that these molecules may exert effects not only at the local level but also systemically [31]. In this study, a significant association was found between the salivary expression profiles of salusin-α and salusin-β and the presence of periodontitis. The findings indicate that these peptides may also be linked to the systemic inflammatory component of periodontal diseases.

There is strong evidence that periodontal diseases are not limited to oral tissues but may also serve as indicators or triggers of systemic inflammation. The association between periodontitis and systemic conditions such as cardiovascular diseases, diabetes, and metabolic syndrome has been demonstrated in numerous studies [1,6,7]. One of the key underlying mechanisms of these associations is the persistence of low-grade systemic inflammation; indeed, it has been reported that periodontal treatments can lead to a reduction in systemic inflammation levels [32,33].

In this context, the involvement of salusin peptides in both local periodontal inflammation and systemic inflammatory response processes makes them potential biomarkers that could serve as a biochemical bridge between periodontal diseases and systemic conditions. In this study, salivary levels of salusin-α and salusin-β were found to be significantly increased in individuals with periodontitis, while only salusin-β was elevated in the gingivitis group. Although salusin-α levels in the gingivitis group appeared lower than in the healthy group, this difference did not reach statistical significance (*p* > 0.05). Therefore, no definitive conclusions can be drawn regarding the suppression of anti-inflammatory mechanisms during gingivitis. However, the increase in salusin-α levels during the periodontitis stage may suggest that the body develops a compensatory or protective response against progressive inflammation. This is consistent with the anti-atherogenic and anti-inflammatory properties of salusin-α. The consistently high levels of salusin-β in both gingivitis and periodontitis cases suggest that this peptide may play a continuous pro-inflammatory role in periodontal tissue destruction.

In the gingivitis and periodontitis groups, a positive, strong, and statistically significant correlation was found between salusin-α and salusin-β levels. This indicates that during the inflammatory process, salusin-α may exhibit an opposite trend, while salusin-β tends to increase in the same direction The high levels observed in the periodontitis group may also reflect an increase in systemic inflammatory activity as the disease progresses [1,4,8].

Considering the results of the ROC analysis, both parameters appear to have potential as discriminative biomarkers between periodontitis and healthy groups; however, the observed discriminatory performance was only moderate (AUC ≈ 0.72–0.73). Given the relatively small sample size and the absence of internal validation (e.g., bootstrap or split-sample approaches), the proposed cut-off values should be regarded as exploratory rather than clinically validated. Therefore, these findings should be interpreted with caution and supported by further analyses and validation studies in larger, independent cohorts.

One of the limitations of the current study is that changes in salusin levels after periodontal treatment were not evaluated; therefore, causal inferences and treatment-related dynamics could not be assessed. Additionally, further studies are needed to investigate salusin-α and salusin-β levels not only in saliva but also in GCF or serum. Another limitation is the inherent difficulty of standardizing saliva as a diagnostic biological fluid, with the lack of control over pre-analytical factors, such as salivary flow rate normalization (pg/mL vs. pg/min) indicating this challenge.

An additional limitation is the significant age disparity among the groups. Given the association of salusins with vascular aging and atherosclerosis, age may have acted as a confounding factor, and the observed differences in salusin levels may not be solely attributable to periodontal status. Therefore, the findings should be interpreted with caution. Although periodontitis is more prevalent at older ages, future studies should use age-matched groups and/or adjust for age in multivariable analyses.

On the other hand, a strength of this study is that, to the authors’ knowledge, no previous study has simultaneously compared salusin-α and salusin-β levels in the saliva samples of patients with gingivitis and periodontitis. This suggests that salusin-α and salusin-β may serve as biomarkers for periodontitis. Thus, it may enable early diagnosis through a non-invasive method before the development of periodontitis, allowing timely initiation of treatment.

For these reasons, future prospective studies with larger sample sizes are needed to fully elucidate the role of salusin peptides in the pathogenesis of periodontal diseases. These studies should investigate the dynamics and molecular mechanisms of these peptides in periodontal diseases and more thoroughly reveal their systemic effects.

## 5. Conclusions

This study showed that salivary salusin-α and salusin-β levels were increased in periodontitis, while only salusin-β was elevated in gingivitis. Accordingly, the first null hypothesis was accepted and the second was rejected. These findings suggest that salusin-β may have a sustained pro-inflammatory role in periodontal disease, whereas salusin-α may be involved in a compensatory response in advanced stages. The positive correlation between the two peptides supports their involvement in periodontal inflammation. Overall, salusin-α and salusin-β may serve as potential non-invasive salivary biomarkers for periodontitis; however, further studies with larger sample sizes are needed to confirm these results.

## Figures and Tables

**Table 1:** Results of one-way ANOVA and post hoc test for sex and age.

	Gingivitis (*n* = 27)	Periodontitis (*n* = 27)	Healthy (*n* = 26)	*p*-Value
Sex n (%)				
Male	10 (37.0%)	11 (40.7%)	6 (23.1%)	0.3597 ^1^
Female	17 (63.0%)	16 (59.3%)	20 (76.9%)	
Age Mean (SD)	29.2 (7.11) ^b^	44.1 (11.38) ^a^	26.2 (6.03) ^b^	<0.0001 ^2^

^1^ Chi-Square *p*-value; ^2^ One-Way ANOVA *p*-value. Lowercase letters (a,b), indicate within row comparisons. Values in the same row with the same lowercase letters are not significantly different according to one-way ANOVA followed by Tukey’s HSD post hoc test. Different letters indicate statistically significant differences between groups (*p* < 0.05).

**Table 2:** Salusin-α (pg/mL) and salusin-β (pg/mL) levels in individuals with gingivitis and periodontitis.

	Healthy (*n* = 26) Mean (±SD)	Gingivitis (*n* = 27) Mean (±SD)	Periodontitis (*n* = 27) Mean (±SD)	*p*-Value
Salusin-α	514.99 (±278.48) ^a,A^	379.00 (±289.86) ^b,A^	870.47 (±720.51) ^a,b,A^	<0.001 For a *p* = 0.024 For b *p* = 0.001
Salusin-β	250.48 (±204.06) ^a,A^	328.67 (±200.05) ^b,B^	651.13 (±501.32) ^a,b,B^	<0.001 For a *p* = 0.000 For b *p* = 0.003
r	0.0780	0.8131	0.8043	
*p*-value	0.7048	0.0001	<0.0001	

Lowercase letters (a, b) indicate within row comparisons, while uppercase letters (A, B) indicate between column comparisons. The same lowercase letters in the row mean statistical difference according to one-way ANOVA (*p* < 0.05). Different uppercase letters in columns indicate statistical difference according to Student’s *t*-test (*p* < 0.05). r, correlation coefficient.

**Table 3:** ROC analysis results.

	AUC	SE	95% CI		*p*-Value	Cutoff	Sensitivity	Specificity
Salusin-α	0.730	0.070	0.594	0.867	0.001	847.28	0.481	0.960
Salusin-β	0.722	0.073	0.579	0.865	0.002	428.18	0.667	0.800

AUC, area under the curve; CI, confidence interval; SE, standard error.

## Data Availability

The data generated in the present study may be requested from the corresponding author.

## Abbreviations

The following abbreviations are used in this manuscript:

MiRNA	microRNA
CAL	Clinical Attachment
BOP	Bleeding on Probing
PD	Probing Depth
PI	Plaque Index
GCF	Gingival Crevicular Fluid
HRP	Horseradish Peroxidase
TBM	Tetramethylbenzidine
IL-1	Interleukin-1
IL-6	Interleukin-6
IL-8	Interleukin-8
IL-18	Interleukin-18
TNF-α	Tumor Necrosis Factor-alpha
ROC	Receiver Operating Characteristic
AUC	Area Under the Curve
ROS	Reactive Oxygen Species
